# Separating the components of an abdominal wall fellowship

**DOI:** 10.1308/rcsann.2022.0058

**Published:** 2022-11-14

**Authors:** ST Adams, M Scott, C West, CJ Walsh

**Affiliations:** ^1^ St Helens and Knowsley Teaching Hospitals NHS Trust, UK; ^2^ Wirral University Teaching Hospitals (WUTH) NHS Foundation Trust, UK

**Keywords:** Ventral hernia, Abdominal wall reconstruction, Education, Training

## Abstract

Complex abdominal wall reconstruction is an emerging subspecialty yet, despite the abundance of abdominal wall hernias requiring treatment and the increasing complexity of this type of surgery, there are few opportunities for surgeons to gain subspecialist training in this field. In this paper we discuss the need for focused training in complex abdominal wall reconstruction, outline some of the problems that may be hindering the availability of such opportunities and propose potential solutions to these issues.

## Introduction

Recent years have seen an increasing interest in the development of specialised abdominal wall reconstruction (AWR) units and for such surgery to become recognised as a subspecialty in its own right.^1,2^ The proponents of such changes cite the increasing complexity of the techniques involved and our enhanced understanding of the requirements and implications of abdominal wall surgery.^2–4^ Several papers have been published that serve as blueprints for those seeking to establish dedicated AWR services, but little has been written about how to go about training the future generations of AWR specialists that would be required to run them.^4–7^ In this article we discuss the issues surrounding specialised AWR training and outline how such training programmes could be designed for maximum educational value.

## Why do we need AWR subspecialists?

Recent estimates suggest that roughly 500,000 ventral hernia repairs are performed annually in both the US and Europe, so it would appear that there are plenty of cases with which budding AWR specialists can expand their operative volume and experience.^8^ However, with such a high caseload there are simply too many ventral hernias requiring surgery for it *not* to remain a procedure within the domain of the generalist. Even the most staunch proponent of sub-specialisation would have to agree that the sheer demand for this type of surgery would necessitate only the most complex cases being managed by a dedicated AWR team.^1,4,5^

The centre of excellence model has been successfully applied across a wide range of clinical fields; however, given the undeniable need for the majority of hernia repairs still to be done by generalists some have argued that a line of distinction needs to be drawn between the terms ‘hernia programme’ and ‘centre of excellence’.^5^ The former can be thought of as a more inclusive version of the latter with its focus being on teamwork and collaboration with non-specialists for the betterment of clinical outcomes globally as opposed to only those outcomes from within the specialist unit itself.^5^

**Figure 1:**
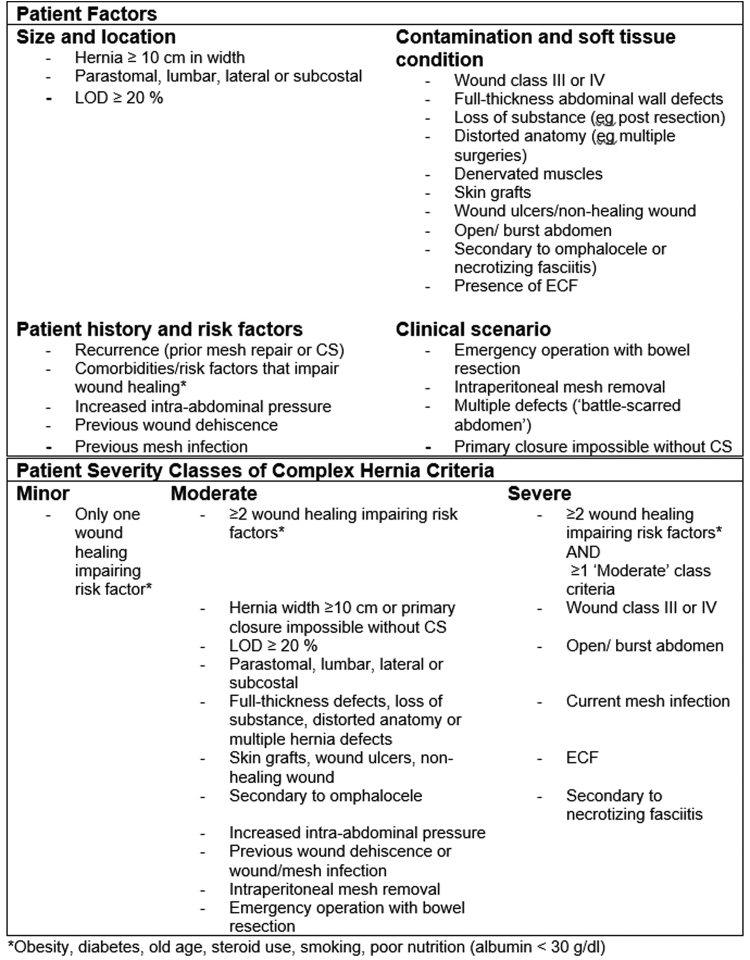
Criteria for complex abdominal wall hernia as defined by Slater *et al*^9^

Patients with complex hernias are liable to be best managed in specialist centres; however, how one defines complexity can vary thus making the identification of referral criteria difficult. Complexity may refer to the hernia itself, the patient, both or simply the likelihood of recurrence or a surgical site occurrence. A useful starting point would be the consensus definition previously described by Slater *et al* in which 22 patient and hernia variables were divided into four separate categories – hernia size and location, contamination/soft tissue condition, patient history/risk factors and clinical scenario – from which three classes of complexity (minor, moderate and severe) were described (Figure 1).^9^ For the majority of patients, in whom few or no risk factors for a ‘complex’ hernia repair are present treatment by a non-specialist is likely to yield comparable outcomes to those of a specialist and thus it would not be justifiable to centralise their care.^1,9^ Conversely, in those patients with risk factors for complexity the evidence suggests that their management by specialist AWR teams would be advantageous both clinically and financially.^10–12^ To aid clinicians in identifying those patients most likely to benefit from referral to a high-volume tertiary centre guidelines have been proposed that advocate for careful patient selection and a multidisciplinary approach to ventral hernia management at the highest tier of complexity.^13^ With regard to training, the mere existence of fellowship programmes has been shown to improve the perioperative outcomes of the units in question.^14,15^

In addition to providing clinical care to the high-risk subgroup of hernia patients, some have argued that specialist AWR teams should also provide a supportive role to the wider surgical community by coordinating research studies and hernia registries and by offering educational opportunities and mentorship.^1,2,4,5^ Such services would address the issues resulting from the decline in operative exposure to hernia repair and AWR that is seen in trainees entering independent practice and ideally would also reduce the number of recurrences ultimately requiring referral to a tertiary centre by enabling less experienced surgeons to optimise their outcomes in line with the national Getting It Right First Time programme.^3,16^

## Why are there so few fellowship programmes in AWR?

General surgical trainees typically experience sub-optimal experience in inguinal hernia repair, but this deficiency has been shown to be rectifiable during fellowship.^3,17^ This is particularly true for the laparoscopic approaches in those undertaking minimally invasive surgery (MIS) programmes.^18^ In contrast to the amount of data regarding inguinal hernia repair there is comparatively little regarding exposure to AWR during either residency or fellowship. Although MIS fellowships provide invaluable experience in many operations around 70% of AWR patients will possess one or more criterion for complexity making the MIS approaches less favourable.^9^ Hence, while there clearly are fellows gaining experience in AWR, they may not be doing so in all the techniques and patient populations required to achieve robust expertise in the field. With roughly 80% of general surgeons in the US and UK undertaking a fellowship after their training we must consider why so few opportunities for AWR-specific training exist.^3^ It is likely that there are multiple underlying reasons:

– Part of the reason for the paucity of fellowship-level training in AWR could result from the fledgling nature of the specialty. If there are few AWR centres at which fellows can obtain high-volume experience, then there are also few places for these fellows to go on to after their training. If there are few opportunities to subspecialise after fellowship, then this may deter candidates from pursuing an AWR-based practice. A lack of candidates could be perceived as a lack of interest, which could in turn lead some to question whether establishing post-residency training in AWR would be viable. Additionally, if the majority of patients are still going to need to have their surgery performed by non-specialists, then this may lead some to believe that focused AWR training is unnecessary.^14,15^ This catch-22 situation makes the creation of an AWR service, and by extension an AWR fellowship, significantly more challenging.
– A second factor could be that AWR is an interface specialty in which both general surgeons and plastic surgeons may play a central role.^19^ An interface specialty is one in which two or more surgical disciplines overlap to share a common area of clinical practice.^20,21^ While the majority of ventral hernia repairs are managed by general surgeons, fellowship-level training in AWR would need to focus on those patients who require more complex repairs for whom a collaborative approach is more appropriate.^19^ Mastery of an interface specialty ideally requires an interface fellowship format.^20,21^ The key aspect of such programmes is that fellows receive training in each of the surgical fields involved thereby expanding the fellow’s knowledge base and operative arsenal beyond that of their original specialty.^21^ Designing such fellowships is challenging since host departments need to be able to provide a trainee-centred educational experience.
– A third contributory factor is funding. In healthcare systems where fee-for-service is standard, specifically in North America, many fellowships are funded from accounts set up to support specific programmes. Fellows bill for their services as surgical assistants with that money going directly into these accounts. The money is then used to pay the fellow a set salary and for the running of the programme – administration, research and academic expenses, etc. Using this model any programme is self-perpetuating so long as the fellows bill for more than their salary and expenses since no external funding is required. In other healthcare systems, such as that of the UK, fellows are an additional expense to the employer, which therefore places the onus on the programmes to prove that the fellowship provides value for money. This in turn incentivises service provision to be favoured over training. Compounding the funding issue is that, despite the long term monetary advantages to improving AWR outcomes, very few grants are available to support clinical development in this area.^22^

## What are the key objectives for a successful AWR fellowship programme?

The Accreditation Council for Graduate Medical Education (ACGME) defines a fellowship as ‘a period of advanced graduate medical education beyond a core residency programme for physicians who desire to enter more specialised practice’.^23^ The most obvious primary objective therefore would be that successful fellows will have acquired both operative and non-operative expertise in the key aspects of AWR beyond that of core training by the time they have completed the programme. A secondary objective would be for both the fellow and the host institution to contribute to AWR-related academic activities such as research and education.

These two goals tie in with the standards required by the European Hernia Society (EHS) for a hernia centre to achieve accreditation.^24^ This provides us with a good starting point from which we can determine the type of facility that could realistically support a fellowship:

– With clinical excellence being the primary objective, any host institution would have to be a high-enough volume centre to be able to sustainably deliver sufficient clinical experience for its fellows to achieve the desired standards in a variety of techniques by its completion.
– Secondly, the host institution would need to possess the infrastructure to be able to coordinate hernia registries, research trials, educational opportunities and the like so that the academic and supportive roles of a hernia programme can be fulfilled.
– Finally, as discussed earlier, the programme should ideally be conducted as a joint venture between general surgery and plastic surgery departments, ideally using an interface fellowship model so that the full range of techniques applicable to AWR can be taught by surgeons from the appropriate specialty.^19^

## How should an AWR fellowship be structured?

This is an area which will be heavily influenced by the model of funding in use and the existing practices and culture of the host facility. In a modular approach the year is split into multiple short-term blocks during which trainees are expected to focus on achieving a specific set of goals before moving on. This is ideal when trying to teach a wide range of subjects and skills which require exposure to a diverse spectrum of clinical experiences. Modular training has been suggested to be more effective than the traditional format of longer periods of service provision and apprenticeship typically seen in Europe.^25^ One major difficulty in adopting a modular design is that it requires centres to seamlessly continue providing the same standard of patient care despite trainees frequently changing blocks and periodically being off service. This is most easily achieved by fellows being supernumerary; however, this would be unappealing for funding bodies looking for consistent returns on their investment.

Regardless of whether a modular or apprenticeship model is adopted, a second consideration is how to provide the high-volume, flexible, trainee-centred educational experience previously discussed. This is where being an interface specialty is advantageous. Few centres will have sufficient volume to provide fellows with five full days worth of AWR experience each week, but by taking a hybridised approach and devoting part of the weekly timetable to gaining experience in an allied surgical discipline then fellows would become more versatile and skilled and, importantly, more employable on their completion. Trauma, MIS, colorectal, bariatrics, surgical oncology – there are numerous fields with significant overlap capable of providing suitable complementary experiences.

A hybridised, modular structure also creates the potential for programmes to become rotational, possibly utilising multiple healthcare facilities in a geographical area. A local syllabus could be bolstered by remote learning with prearranged and agreed exposure to other units. Such remote attendance at other units might not only benefit the fellow but could conceivably be of value to their host unit by cross fertilisation of ideas and practices and by facilitating academic collaboration.

## What should be part of an AWR fellowship syllabus?

**Figure 2:**
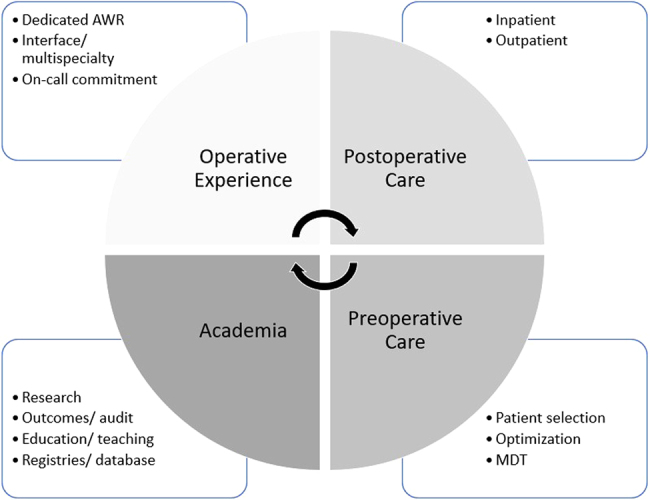
Global objectives of an abdominal wall reconstruction (AWR) fellowship

When devising a syllabus for subspecialty training, one must assume that the appropriate levels of ability have already been achieved in those areas common to all surgical disciplines and generic in a trainee’s background specialty. Additionally, one must accept that any list of educational goals is liable to be controversial. Similarly, it is impossible for such lists to be comprehensive without being impractically long. These discrepancies are due to a variety of influences such as clinical background, local practices, career intentions, health service culture, accessibility of services and patient expectations (Figure 2).

**Figure 3:**
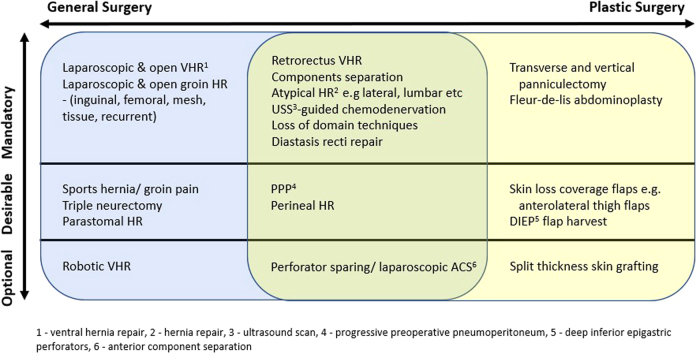
Suggested procedures to be included on abdominal wall reconstruction (AWR) fellowship syllabus. Techniques more relevant to general surgery are positioned on the left whereas those more associated with plastic surgery are positioned on the right; the central area represents the common ground between the two specialties.

It should be emphasised that the horizontal alignments of the procedures shown in Figure 3 should not be seen as exclusive, as this would be contrary to the ethos of an interface fellowship. If, for example, a fellow from a general surgery background has the motivation and opportunity to become proficient in panniculectomy and abdominoplasty, then this should be facilitated. The distinctions between the three vertical tiers are somewhat arbitrary, but broadly speaking for anything mandatory a fellow would be expected to be nearing or beyond the end of their learning curve by the completion of their fellowship. Those in the desirable category are procedures in which competence may be advantageous from a future scope-of-practice point of view but they are not necessarily fundamental to be considered an expert in AWR.

**Figure 4:**
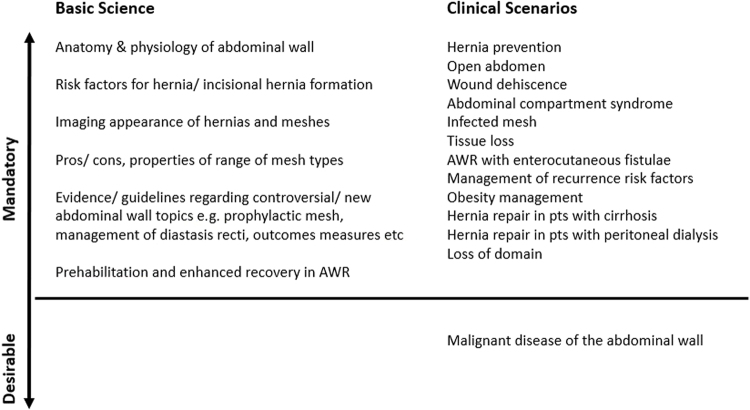
Essential topics in abdominal wall reconstruction (AWR)

As with Figure 3, the list of essential topics to be learnt during an AWR fellowship (Figure 4) is inevitably going to be contentious. The desired standard of knowledge or understanding of the topics shown should be greater than that expected of a generalist such that one would be able to provide advice or clarification if requested. This ties in with the supportive and mentorship roles of a hernia programme as discussed previously.

Active participation in regular AWR multidisciplinary team (MDT) meetings should be encouraged to familiarise fellows with the decision-making processes underlying the management of those patients whose clinical situations fall into the scenarios shown on the right of Figure 4.

## How could programmes confirm that standards are being met?

**Figure 5:**
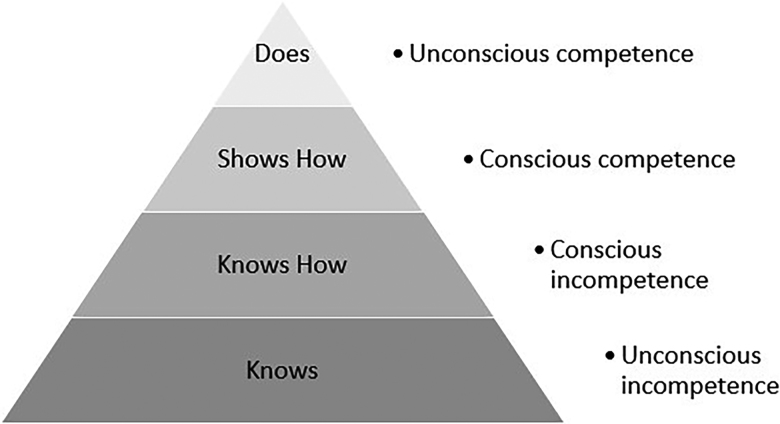
Miller’s triangle^26^

Fellowship training can be thought of as a period in which clinicians make the transition from the supervision and active observation of residency to simply doing the job. It provides an opportunity to ascend from conscious competence to unconscious competence at the apex of Miller’s triangle (Figure 5).^26^

The learning curves for laparoscopic and open inguinal hernia repairs range between 50–100 and 40–64 respectively, numbers which should be easily surpassable during a single year of fellowship.^11,17,27–29^ For the majority of the operations in Figure 3 the learning curves have not been investigated. Data from the National Consultant Information Programme (NCIP) show that the median number of incisional hernia repairs between October 2018 and September 2021 inclusive was 5 per general surgeon with the 95^th^ centile being only 26.^30^ Over the same time period the median number of such operations per hospital is roughly 120.^30^ By concentrating complex AWR in a smaller number of high-volume centres the learning curves for the necessary techniques could easily be studied and subsequently used to guide future training requirements at the specialist registrar level. However, although increased operative volume has been repeatedly shown to result in improved outcomes, it would be remiss of a training programme to rely solely on logbook numbers and fellowship duration to assess competence.^31,32^ Several validated, objective means of assessing operative ability are available. Formative use of such assessment tools should be encouraged at pre-determined intervals throughout fellowship in order to identify areas on which to focus ongoing training needs.

The assessment of knowledge at fellowship level is more problematic, and little has been written on this topic. Although advanced abdominal wall surgery examinations do exist, such as the Fellow of the European Board of Surgery Abdominal Wall Section (FEBS AWS), they are primarily aimed at surgeons with established practices.^33,34^ The syllabus for such exams certainly have merit in terms of guiding fellowship education, and achievement of the required standard should be feasible for the majority of post-FRCS fellows.^34^ However, it could be argued that unless appointment into a substantiative post becomes reliant on the possession of post-FRCS qualifications, then to mandate an additional formal test of knowledge might deter applicants who would otherwise be interested in pursuing an AWR fellowship. Although imperfect, determining whether or not a fellow has acquired an acceptable degree of specialist knowledge will therefore likely remain a subjective judgement call for the foreseeable future.

## Conclusion

In this paper we have outlined the reasons why AWR is worthy of specialist training and what some of the potential barriers to this training may be. The authors believe that while the goals and format of any such training will be inherently controversial, the clinical need for AWR specialisation is sufficiently great for solutions to these barriers to be sought. It is the authors’ hopes that this paper will prompt discussion in the surgical community regarding the establishment of recognised specialist clinical units and that this paper will serve as a blueprint for the formation of further subspecialist training opportunities.

## Conflicts of interest/Competing interests

The authors confirm that they have no conflicting or competing interests that may inappropriately bias this work. STA and CW have nothing to declare. MS has received consulting fees from Allergan Aesthetics and Bard, honoraria for lectures from Bard and support for attending meetings from Bard. CJW has received honoraria for lectures and support for attending meetings from Gore and Cook and has accepted a gift of an AWR textbook on behalf of the Department of Surgery at Wirral University Teaching Hospitals NHS Foundation Trust.
